# Infliximab-Induced Vasculitis in a Rheumatoid Arthritis Patient: A Comprehensive Case Report

**DOI:** 10.7759/cureus.82467

**Published:** 2025-04-17

**Authors:** Thanda Aung, Mia Celestin

**Affiliations:** 1 Rheumatology, University of California Los Angeles David Geffen School of Medicine, Los Angeles, USA

**Keywords:** infliximab, lekocytoclastic vasculitis, rheumatoid arthritis, skin ulcer, tnf inhibitor, tumor necrosis factor-α(tnf-α) inhibitor, vasculitis

## Abstract

Tumor necrosis factor (TNF) inhibitors are widely used biologics in the management of rheumatoid arthritis (RA), but they can occasionally induce paradoxical inflammatory manifestations. We present a case of a 28-year-old female patient with well-controlled rheumatoid arthritis who developed leukocytoclastic vasculitis (LCV) secondary to infliximab therapy. Despite initial treatment with alternative biologics and corticosteroids, her condition required escalation to rituximab therapy. Histopathological examination confirmed leukocytoclastic vasculitis involving vessels in the deep dermis. After 10 months of rituximab treatment, the patient experienced complete resolution of the vasculitis. This case highlights the importance of recognizing rare but significant cutaneous adverse effects of TNF inhibitors and illustrates the challenges in management when conventional therapy proves ineffective. Clinicians should maintain vigilance for paradoxical inflammatory responses when treating patients with biologic agents and consider appropriate therapeutic alternatives when such complications arise.

## Introduction

Rheumatoid arthritis (RA) is a chronic autoimmune disease characterized by synovial inflammation and progressive joint destruction. The introduction of biologic agents, particularly TNF inhibitors, has revolutionized RA management. These medications effectively suppress inflammation by blocking the pro-inflammatory cytokine TNF-alpha, resulting in significant clinical improvements for many patients [1].

However, TNF inhibitors have been associated with various paradoxical immune-mediated adverse events, including cutaneous manifestations [2]. While psoriasiform eruptions are well-documented, leukocytoclastic vasculitis (LCV) is a less common but potentially serious complication. LCV is a small-vessel vasculitis characterized by neutrophilic infiltration, leukocytoclasia (nuclear debris), and fibrinoid necrosis of vessel walls. It typically presents as palpable purpura, predominantly affecting the lower extremities [3].

The mechanism underlying TNF inhibitor-induced vasculitis remains incompletely understood. Current theories suggest that TNF blockade may lead to dysregulation of cytokine networks, formation of immune complexes, or production of autoantibodies that trigger vascular inflammation [4]. This paradoxical effect highlights the complex immunomodulatory actions of these biologics.

We report a case of TNF inhibitor-associated LCV in a young female with RA, emphasizing the diagnostic challenges and management strategies for this rare complication.

## Case presentation

A 28-year-old female with a history of rheumatoid factor-positive (RF+) and anti-cyclic citrullinated peptide-positive (CCP+) RA presented to the rheumatology clinic with a several-month history of a rash on her left shin that had recently become painful and edematous. Her RA had been well-controlled for the past two to three years with infliximab administered every eight weeks, in combination with hydroxychloroquine.

At presentation, the patient denied fever, chills, or nausea. She had no prior history of rashes and no other underlying health issues. She denied any new vaccines, medications, sick contacts, or recent travel. Physical examination revealed an erythematous, palpable rash on the left shin with associated edema and tenderness.

Laboratory investigations showed a complete blood count with slightly elevated red blood cell count, hemoglobin, and hematocrit, however, her comprehensive metabolic panel was normal (Table 1). They also revealed a positive antinuclear antibody (ANA) with a titer of 1:640 (homogeneous and speckle pattern), a slightly elevated erythrocyte sedimentation rate (ESR) of 31 mm/hr, and C-reactive protein (CRP) of 1 mg/dL. Her complement levels, anti-double-stranded DNA (dsDNA), angiotensin-converting enzyme, and 1,25-dihydroxy-vitamin D were unremarkable. Anti-neutrophil cytoplasmic antibodies (ANCA) and cryoglobulin were negative. Hypercoagulability panel (prothrombin, activated partial thromboplastin time, fibrinogen, protein C, protein S, antithrombin, homocysteine, DRVVT (dilute Russell's viper venom time), antiphospholipid antibody syndrome (APS) antibodies were normal, though D-dimer was elevated. All workups for potential infections, including hepatitis B, hepatitis C, *Mycobacterium tuberculosis*, and testing for streptococcal and aspergillus antigens, were negative. Testing for antibodies to infliximab was also negative (Table 2).

**Table 1:** Comprehensive metabolic panel and complete blood count

Pertinent Lab Data	Patient's Lab Values	Reference Range
Sodium (mmol/L)	137	135 - 146
Potassium (mmol/L)	3.8	3.6 - 5.3
Chloride (mmol/L)	104	96 - 106
Total CO2 (mmol/L)	22	20 - 30
Anion gap (mmol/L)	11	8.0 - 19.0
Glucose (mg/dL)	81	65 - 99
Estimated glomerular filtration rate (mL/min/1.73m2)	>89	> 89
Creatinine mg/dL	0.73	0.60 - 1.30
Urea nitrogen (mg/dL)	16	7.0 - 22.0
Calcium (mg/dL)	9.5	8.6 - 10.3
Total protein (g/dL)	7.7	6.1 - 8.2
Albumin (g/dL)	4.4	3.9 - 5.0
Bilirubin, total (mg/dL)	0.5	0.1 - 1.2
Alkaline phosphatase (U/L)	82	37 - 113
Aspartate aminotransferase (U/L)	27	13 - 47
Alanine aminotransferase (U/L)	43	8.0 - 64.0
White blood cell count (1/uL)	8.81	4.16 - 9.95 x10E3
Red blood cell count (1/uL)	5.15	3.96 - 5.09 x10E6
Hemogloblin (g/dL)	15.7	11.6 - 15.2
Hematocrit (%)	47.5	34.9 - 45.2
Mean corpuscular volume (fL)	92.2	79.3 - 98.6
Mean corpuscular hemoglobin (pg)	30.5	26.4 - 33.4
Mean corpuscular hemoglobin concentration (g/dL)	33.1	31.5 - 35.5
Red cell distribution width - SD (fL)	42.7	36.9 - 48.3
Red cell distribution width - CV (%)	12.6	11.1 - 15.5
Platelet count, auto (1/uL)	340	143 - 398 x10E3
Mean platelet volume (fL)	10.5	9.3 - 13.0
Absolute nucleated red blood cell count (1/uL)	0	0.00 - 0.00 x10E3
Neutrophil antibodies (1/uL)	6.23	1.80 - 6.90 x10E3
Absolute lymphocyte count (1/uL)	1.57	1.30 - 3.40 x10E3
Absolute monocyte count (1/uL)	0.64	0.20 - 0.80 x10E3
Absolute eosinophil count (1/uL)	0.34	0.00 - 0.50 x10E3
Absolute basophil count (1/uL)	0.06	0.00 - 0.10 x10E3
Absolute immature granulocyte count (1/uL)	0.02	0.00 - 0.04 x10E3

**Table 2:** Other pertinent lab results ANA: antinuclear antibody; ESR: erythrocyte sedimentation rate; C3: complement 3; C4: complement 4; dsDNA Ab EIA: double-stranded DNA antibody enzyme immunoassay; nDNA Ab IFA: native DNA indirect fluorescent antibody; C-ANCA: cytoplasmic antineutrophil cytoplasmic antibody; P-ANCA: perinuclear antineutrophil cytoplasmic antibody; Protein 3 Ab: protein 3 antibody; Myeloperoxidase Ab: myeloperoxidase antibody; DRVVT: dilute Russell's viper venom time; MTB-Quantiferon-Gold ELISA: *Mycobacterium tuberculosis* Quantiferon-Gold enzyme-linked immunosorbent assay

Pertinent Lab Data	Patient's Lab Values	Reference Range
ANA Ab titer	1:640	<1:40
ESR (mm/hr)	31	<=25
C-reactive protein (mg/dL)	1	<0.8
C3 (mg/dL)	130	86 - 175
C4 (mg/dL)	19	10.0 - 40.0
dsDNA Ab EIA (IU/mL)	<=200	<=200
nDNA (Crithidia) Ab IFA (titer)	<1:10	<1:10
C-ANCA (titer)	<1:20	<1:20
P-ANCA (titer)	<1:20	<1:20
Protein-3 Ab (CU)	<20.0	<20.0
Meyloperoxidase Ab (CU)	<20.0	<20.0
Prothrombin (seconds)	13.2	11.5-14.4
Activated partial thromboplastin Time (seconds)	29.3	24.4-36.2
Fibrinogen (mg/dL)	468	235-490
D-dimer (ug/mL)	0.79	<0.60
Protein C (%)	128	70-130
Protein S (%)	122	57-131
Antithrombin III activity (%)	118	80-120
Homocysteine, total (mcmol/L)	6	<=15
DRVVT	Negative	Negative or Positive
Beta-2-glycoprotein IgA (SAU)	<10	<=20
Beta-2-glycoprotein IgG (SGU)	<10	<=20
Beta-2-glycoprotein IgM (SMU)	<10	<=20
Cardiolipin IgA (CU)	<20	<=20
Cardiolipin IgG (CU)	<20	<=20
Cardiolipin IgM (CU)	<20	<=20
Hepatitis B	Nonreactive	Nonreactive
Hepatitis C	Nonreactive	Nonreactive
MTB-Quantiferon-Gold ELISA	Negative	Negative
Antibodies to infliximab, ATI (U/mL)	<3.1	<3.1

**Figure 1:**
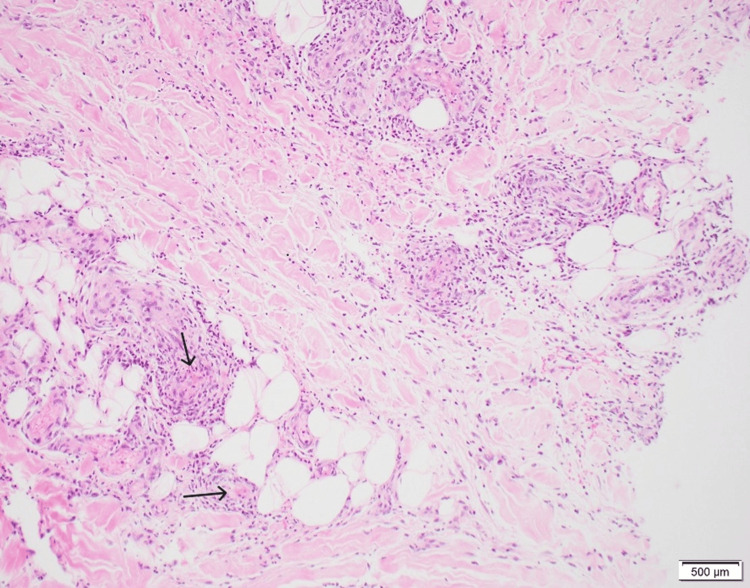
Skin biopsy, CD34 immunohistochemical stain showing leukocytoclastic vasculitis, magnification 100X

**Figure 2:**
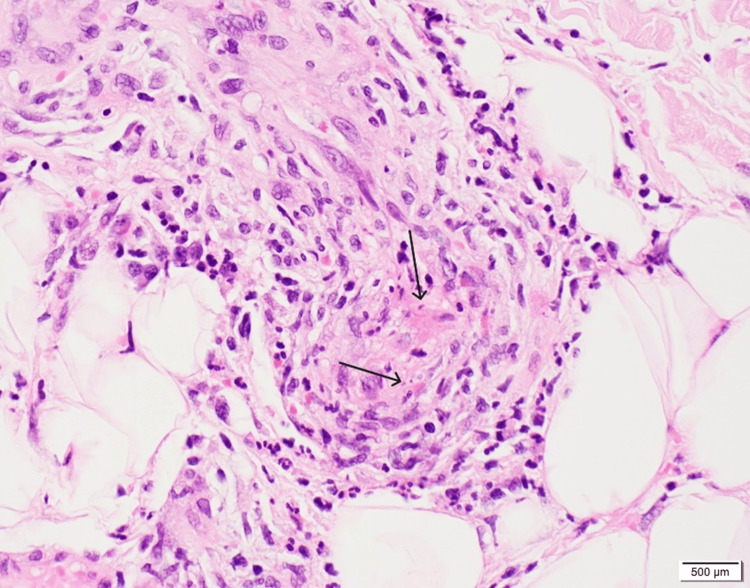
Skin biopsy, CD34 immunohistochemical stain showing leukocytoclastic vasculitis, magnification 400X

Over several weeks, the rash continued to spread. A punch biopsy of the affected skin was performed, confirming the diagnosis of leukocytoclastic vasculitis involving vessels in the deep dermis and subcutis. The CD34 immunohistochemical stain was positive, highlighting vascular structures (Figures 1-2). Given that the patient's RA had been well-controlled for years and there was no evidence of current infection or malignancy, the condition was diagnosed as anti-TNF induced vasculitis.

**Figure 3:**
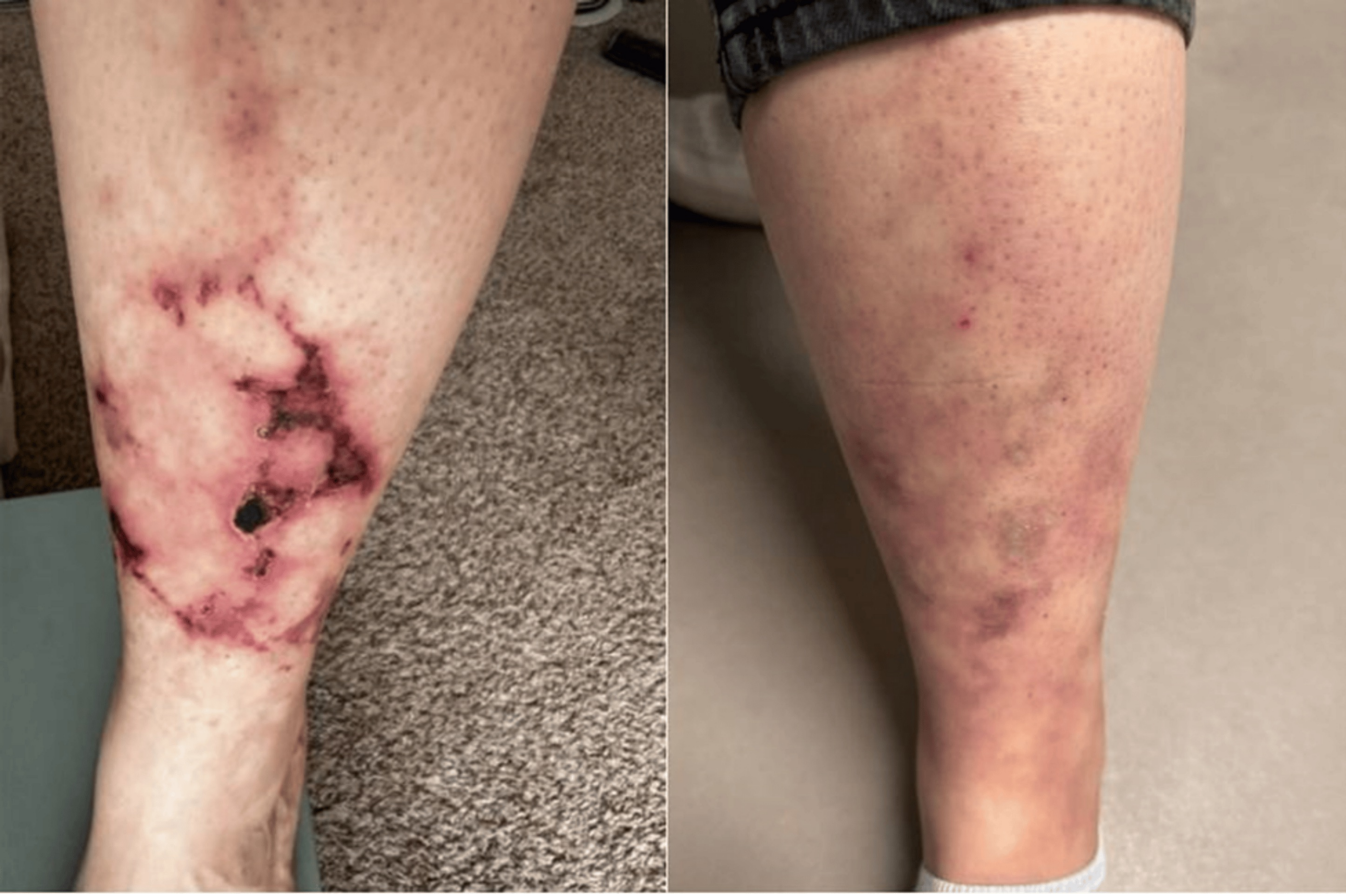
Left lower extremity rash and ulceration, before ritiximab infusions (left side) vs resolution 10 months after rituximab infusions (right side)

Following diagnosis, the patient was switched from infliximab to tocilizumab at a dose of 522 mg (4 mg/kg body weight) every eight weeks. She was also started on prednisone (40 mg/day), colchicine 0.6 mg daily, and topical dapsone 5% gel. Despite this treatment regimen, her condition did not respond significantly, and she experienced worsening pain around the rash area with progression to ulceration of the left lower extremity (Figure 3).

Due to the progressive nature of the LCV exceeding four weeks' duration and ulceration, systemic therapy was escalated to include Rituximab infusions every six months. Additionally, her treatment regimen was modified. Topical dapsone 5% gel was discontinued to reduce the risk of methemoglobinemia, and oral dapsone 50 mg daily was initiated. Two months after the initiation of Rituximab, her rashes had started to improve. The prednisone taper was done over a six-month period.

At 10 months after initiation of rituximab therapy, the patient's LCV had completely resolved with no residual pain (Figure 3). She continues maintenance therapy, rituximab infusions 1000 mg, 2 doses, every 6 months, and hydroxychloroquine. Both colchicine and oral dapsone were discontinued.

## Discussion

There are multiple potential causes for cutaneous small to medium vessel vasculitis, including infection, malignancy, and autoimmune conditions such as rheumatoid arthritis. In patients with RA, vasculitis typically occurs as a rare complication in approximately 1-5% of cases, usually in the setting of long-standing, poorly controlled disease characterized by joint deformities and persistently abnormal immunological and inflammatory markers [3,5].

However, our patient's presentation was atypical for primary RA-associated vasculitis. Her RA had been in remission for 2-3 years prior to the development of the rash, and her inflammatory markers were only slightly elevated following the diagnosis of LCV. These factors, combined with the temporal relationship to Infliximab therapy, pointed toward a medication-induced vasculitis.

TNF inhibitors are commonly used biologics for patients with RA as they suppress TNF-mediated inflammation. While these agents are known to cause various skin manifestations such as psoriasiform eruptions [2,6], their association with leukocytoclastic vasculitis is less frequently reported but increasingly recognized. The exact mechanism of TNF inhibitor-induced vasculitis is not fully understood; however, it is hypothesized that when apoptosis is induced, there is an aggregation of antigens and overproduction of autoantibodies, triggering an immune response that leads to vascular inflammation and subsequent rash [7].

Paradoxical adverse events (PAEs) are conditions that develop during biological agent therapy, contrary to the drug's expected therapeutic effect. They primarily occur with TNF-α agents and involve dermatological, intestinal, and ophthalmic manifestations. True PAEs include psoriasis, Crohn's disease, and hidradenitis suppurativa, while borderline cases encompass conditions like uveitis, vasculitis, and sarcoidosis. Potential explanations include cytokine imbalance, immunological variations, and shifts in immune system profiles [4].

Since various rashes can resemble each other clinically, a skin biopsy remains the gold standard for accurate diagnosis of LCV. Histopathologically, LCV is characterized by neutrophilic infiltration around blood vessels, leukocytoclasia (nuclear dust), fibrinoid necrosis of vessel walls, and extravasation of erythrocytes [8-9]. In our patient, CD34 immunohistochemical staining further highlighted the affected vascular structures, confirming the diagnosis.

Management of drug-induced LCV typically involves discontinuation of the offending agent and initiation of immunosuppressive therapy. First-line treatment often includes corticosteroids, with the addition of steroid-sparing agents in refractory cases [8,10]. In our patient, the combination of high-dose prednisone and rituximab infusions proved effective after initial treatment with Tocilizumab, colchicine, and dapsone showed a limited response.

This case illustrates several important clinical considerations. First, it highlights the importance of recognizing paradoxical adverse effects of biologic therapies, even in patients with well-controlled underlying disease. Second, these adverse effects of biologic therapies, even in patients with well-controlled underlying diseases, demonstrate the need for careful medication selection in patients with multiple contraindications to standard therapies. Finally, it underscores the potential effectiveness of B-cell depletion therapy with rituximab in cases of refractory LCV.

The use of rituximab in our case was particularly noteworthy. While this B-cell-depleting agent is traditionally used for RA, its effectiveness in treating vasculitis has been well documented [10]. The resolution of our patient's LCV with rituximab suggests that B-cell-mediated processes may play a significant role in the pathogenesis of drug-induced vasculitis.

Despite testing negative for antibodies to Infliximab, drug-induced vasculitis remained the most likely diagnosis based on the temporal relationship, clinical presentation, and exclusion of other causes. This highlights that immune mechanisms other than antibody formation against the biologic agent itself may be involved in the pathogenesis of TNF inhibitor-induced vasculitis.

## Conclusions

Cutaneous vasculitis in RA patients treated with TNF inhibitors presents a significant diagnostic and therapeutic challenge, requiring careful differential diagnosis and individualized management. While biologic therapies have revolutionized RA treatment, this case demonstrates their potential complications, highlighting the importance of vigilant monitoring and alternative approaches like rituximab for refractory cases, though further research is needed to understand the mechanisms of TNF inhibitor-induced vasculitis and develop optimal management strategies for this rare complication.
